# Effects of Aloe Vera in the Treatment of Oral Ulcers: A Systematic Review and Meta-Analysis of Randomised Controlled Trials

**DOI:** 10.3290/j.ohpd.b3666483

**Published:** 2022-12-12

**Authors:** Hang Zou, Zengjing Liu, Zhi Wang, Juan Fang

**Affiliations:** a Dentist, Hospital of Stomatology, Guanghua School of Stomatology, Sun Yat-Sen University, Guangzhou, China; Guangdong Provincial Key Laboratory of Stomatology, Guangzhou, China. Designed the meta-analysis protocol, participated in the data extraction and analysis, drafted the manuscript.; b Master Student, School of Information and Management, Guangxi Medical University, Nanning, China. Participated in the data extraction and analysis, drafted the manuscript.; c Professor, Hospital of Stomatology, Guanghua School of Stomatology, Sun Yat-Sen University, Guangzhou, China; Guangdong Provincial Key Laboratory of Stomatology, Guangzhou, China. Supervised the work, revised the manuscript.; d PhD Student, Hospital of Stomatology, Guanghua School of Stomatology, Sun Yat-Sen University, Guangzhou, China; Guangdong Provincial Key Laboratory of Stomatology, Guangzhou, China. Supervised the work, participated in the data extraction and analysis, drafted and revised the manuscript.; * Contributed equally to the manuscript.

**Keywords:** Aloe vera, clinical therapeutic effect assessment, meta-analysis, randomised controlled trials (RCTs), treatment of oral ulcers

## Abstract

**Purpose:**

To analyse the effects of aloe vera on the treatment of oral ulcers.

**Materials and Methods:**

Relevant studies were identified from PubMed, Embase, ClinicalTrials.gov, SinoMed, Web of Science and Cochrane Library. Randomised controlled trials (RCTs) involving aloe vera (AV) in the treatment of oral ulcers were included. The data were extracted and pooled for meta-analysis to compare the clinical outcomes of patients with oral ulcers in terms of treatment effect, the size of ulcers, Visual Analogue Scale (VAS) for pain, and therapy duration. The standardised mean differences (SMD) with 95% confidence intervals (CI) were determined for the main outcomes, heterogeneity was analysed using the I^2^ test, and the studies’ risk of bias was evaluating using the Cochrane risk of bias assessment tool.

**Results:**

The study included 9 trials with a total of 847 participants. Seven trials were included in the meta-analysis. The results indicated no statistically significant differences in pain scores as assessed by the VAS (I^2^ = 95%, p = 0.89; SMD: -0.12, 95% CI: -1.84, 1.60) and size of ulcers (I^2^ = 88%, p = 0.60; SMD: -0.29, 95% CI: -1.39, 0.81). Clinical efficacy of the aloe vera group was better compared to control group (I^2^ = 89%, p = 0.007; RR: 2.25, 95% CI: 1.25, 4.06). Therapy duration was statistically significantly lower following AV gel application in two of the studies (I^2^ = 0%, p < 0.001; SMD: -1.32, 95% CI: -1.84, -0.79). Considering the results in a systematic manner, AV accelerated tissue epithelialisation and the wound-healing process.

**Conclusion:**

Compared with other interventions, aloe vera has a better therapeutic effect and shorter healing time. It is comparable with other interventions in relieving pain and reducing ulcer size, but it has higher safety and almost no side effects.

Oral ulcers are a disease of the oral mucosa, described as a loss of mucous substance of the mouth, showing local excavation of the surface, resulting from sloughing of inflammatory necrotic tissue. Various oral ulcers reduce the quality of life of affected people, such as recurrent aphthous stomatitis, traumatic oral ulceration and mucositis caused by radiation. Recurrent aphthous stomatitis (RAS) is a common disorder; the cardinal symptoms are single or multiple ulcers with clear boundaries, inflammatory exudate and pain.^1,6,23^ Traumatic oral ulceration is a side effect of orthodontic treatment frequently found in the mucous membranes of the gums and the inside of the cheeks accompanied by symptoms similar to RAS; it is also a side-effect of radiation therapy.^15^ Although traumatic oral ulceration and mucositis induced by radiation can disappear or diminish by stopping radiation, oral ulcers still seriously impact the daily quality of life of patients. Specific curative management is still lacking, despite the availability of multiple medications which are locally applied or systemically administered, aiming reduce pain and inflammation, while promoting the healing of oral ulcers.^17,23^ Therefore, it is necessary to develop an effective agent for the treatment of oral ulcers.

Substantial evidence has shown that oral health and disease are intricately connected to the oral microbiome, which in turn is affected by various factors, including genetics, environment, and systemic condition. Dietary habits, as an important environmental factor, have a key influence on the maintenance of oral hygiene and the occurrence and progression of oral diseases. Natural herbal products, as food supplements, are used world-wide and are claimed to foster physical fitness and prevent disease.^12,21,22^ Aloe vera is characterised as a medicinal plant with various beneficial properties, and has gained considerable attention in clinical research. It is reported to be biochemically very complex, containing numerous substances, such as minerals, enzymes, sugars, anthraquinones, lignin, saponins, sterols, amino acids and salicylic acid, with pleiotropic functions having anti-inflammatory, antibacterial, antioxidant, radiation protective, immune-boosting and hypoglycemic properties.^19,25^ In dentistry, aloe vera has also been utilised with remarkable effects. Some studies examined the use of aloe vera for treatment of extraction socket and endodontic medication, or maintainance of oral hygiene through reducing dental plaque.^2,24^ Moreover, aloe vera has also been shown to play an important role in the management of oral lesions, such as oral lichen planus, oral submucous fibrosis, radiation-induced mucositis, burning mouth syndrome and xerostomia.^5,16,20^

To investigate possible advantages of aloe vera in the clinical treatment of oral ulcers, the purpose of this study was to systematically review the effect of aloe vera on oral ulcers, based on the findings of clinical trials.

## Methods

We designed and reported this systematic review and meta-analysis according to the guidelines of the preferred reporting items for systematic reviews and meta-analyses (PRISMA).

### Search Strategy

A systematic search was performed of electronic medical databases, including PubMed, Embase, SinoMed, Web of Science and Cochrane Library from their inception to December 10, 2021, without any restriction for time or language. Medical subject headings (MeSH) combined with text words were applied to identify qualified articles. The complete search words for PubMed were: “(((“”Oral Ulcer” ”[Mesh]) OR (((((((Oral Ulcers[Title/Abstract]) OR (Ulcer, Oral[Title/Abstract])) OR (Ulcers, Oral[Title/Abstract])) OR (Mouth Ulcer[Title/Abstract])) OR (Mouth Ulcers[Title/Abstract])) OR (Ulcer, Mouth[Title/Abstract])) OR (Ulcers, Mouth[Title/Abstract]))) AND ((“”Aloe””[Mesh]) OR ((aloe[Title/Abstract]) OR (aloe vera[Title/Abstract])))) AND (randomized controlled trial[Publication Type] OR randomized[Title/Abstract] OR placebo[Title/Abstract])”,,,”(“”Oral Ulcer””[MeSH Terms] OR (“”oral ulcers””[Title/Abstract] OR “”ulcer oral””[Title/Abstract] OR “”ulcers oral””[Title/Abstract] OR “”mouth ulcer””[Title/Abstract] OR “”mouth ulcers””[Title/Abstract] OR ((“”Ulcer””[MeSH Terms] OR “”Ulcer””[All Fields] OR “”ulcerate””[All Fields] OR “”ulcerated””[All Fields] OR “”ulcerates””[All Fields] OR “”ulcerating””[All Fields] OR “”ulceration””[All Fields] OR “”ulcerations””[All Fields] OR “”ulcerative””[All Fields] OR “”Ulcers””[All Fields] OR “”ulcer s””[All Fields] OR “”ulcerous””[All Fields]) AND “”Mouth””[Title/Abstract]) OR “”ulcers mouth””[Title/Abstract])) AND (“”Aloe””[MeSH Terms] OR (“”Aloe””[Title/Abstract] OR “”aloe vera””[Title/Abstract])) AND (“”randomized controlled trial””[Publication Type] OR “”randomized””[Title/Abstract] OR “”placebo””[Title/Abstract])”.

### Study Selection

Two authors (HZ and ZJL) independently and carefully reviewed non-duplicate records found in the initial search. The articles were excluded if they did not meet the inclusion criteria, or fulfilled the exclusion criteria. Furthermore, the full text of eligible articles was reviewed and any disagreements were discussed until a consensus was met.

### Inclusion Criteria

Original articles which investigated the effects of aloe vera and/or its extracts on oral ulcers were included only if the following criteria were met: (a) a randomised controlled trial design was employed; (b) at least two groups were included: an intervention group with aloe vera or aloe-vera extract, and a control group with placebo or other treatments, except medications based on aloe vera; (c) patient groups consisted of both children and adult patients with oral ulcers as a main condition.

### Exclusion Criteria

The following criteria were applied to exclude irrelevant records: (a) studies which used a mixture of aloe vera with other medicines and aloe vera was not the main component; (b) uncontrolled studies; (c) nonrandomised controlled trials; (d) duplicate data; and (e) reviews, letters, editorial articles, study protocols, animal experiments, or case reports.

### Data Extraction

The following information was extracted from the full-text of eligible papers and reported in Table 1, using a predesigned form: publication information including author’s last name, publication date, details of clinical trial including study design, sample size, gender, age (in years), intervention (dosage and type of aloe vera or control group), study duration, main outcomes with emphasis on the effects on pain, effects on ulcer size and healing time.

**Table 1:** Characteristics of reviewed studies

First author (year)	Country	Study design	Sample size	Gender (male/female)	Age (mean or range of age) (T/C)	Dosing regimen		Study duration	Outcomes
						Aloe vera gel (T)	Control( C)		
Garnick et al. (1998)	USA	RCT	34	10/24	35	Aloe vera gel	Carrier gel only	3 months	Therapy duration, mean interval, VAS score for pain, size of ulcers
Duan et al. (2008)	China	RCT	64	34/30	38	Aloe chitosan oral compound membrane	Iodine glycerin	5 days	Therapy duration, VAS score for pain, therapeutic effect
Babaee et al. (2012)	Iran	RCT	49	22/18	27.95 ± 7.96/ 29.25 ± 8.48	Freshly purified leaf juice extract of A.V. gel with 1.6% dry remnant and a density of 1.01 g/ml was used to prepare a 2% oral gel using sterile lubricant gel	Lubricant gel containing 2% normal saline	14 days	VAS score for pain, size of ulcers, VAS score for burning, erythema and exudation scores
Bhalang et al. (2013)	Thailand	RCT	330	–	–	0.5% acemannan i n Carbopol 934 PNF	(A) 0.1% triamcinolone acetonide pure Carbopol 934 PNF (C) 0.5% acemannan in Carbopol 934 PNF	7 days	Size of ulcers, VAS score for pain, skin patch test, blood test
Deng et al. (2013)	China	RCT	80	40/40	5–68	Aloe vera gel	Superior sore throat powder 0.2 g	7 days	Therapy duration, pain
Mansour et al. (2014)	Saudi Arabia	RCT	90	38/52	31.7 ± 8.4	(A) mucoadhesive gel with aloe vera (B) mucoadhesive gel with myrrh extract	Plain mucoadhesive gel	6 days	Size of ulcers, VAS score for pain, erythema and exudation scores
Sahebjamee et al. (2015)	Iran	RCT	26	–	55.38 ± 17.99 / 59.31 ± 15.03	Aloe vera mouthwash containing pure Aloe vera gel 0.0009% Brilliant Blue dye, 0.0006% tartrazine yellow dye and 2. 0.15% benzydamine	0.15% benzydamine mouthwash	14 weeks	WHO system of mucositis grading, Interval from start of RT to mucositis onset by day, Interval from start of RT to maximum mucositis grade by day
Giroh et al. (2019)	India	RCT	34	17/17	20–50	5 mg of aloe vera gel	Triamcinolone acetonide 0.1% (kenacort oral paste)	7 days	Size of ulcers,VAS score for pain,VAS score for burning
Leiva-Cala et al. (2020)	Japan	RCT	140	51/89	26.1 ± 11.1	80% of aloe vera combined with carbopol, cross-linked acrylic acid hydrophilic polymer (HPMC), and ascorbic acid	The CHX formulation used is commercially available as 0.12% Lacer Bioadhesive Gel	30 days	Traumatic oral ulceration (TOU), plaque (PI), gingival index (GI), gingival bleeding index (GBI)

### Quality Assessment and Risk of Bias

Two authors assessed the risk of bias for each of the included studies based on the Cochrane risk of bias assessment tool. This tool covers six domains of bias: (a) selection bias; (b) performance bias; (c) detection bias; (d) attrition bias; (e) reporting bias; and (f) other bias. According to the specific criteria for each domain, the risk of bias is grouped as low risk (+), unclear (?), or high risk (-). Any discrepancies were resolved by consensus in the presence of a third investigator.^13^ Due to the limited number of studies included in the meta-analysis, no publication bias or sensitivity tests were conducted.

### Statistical Analysis

If more than one experimental group was reported in some clinical trials, each experimental group vs control group was selected for the analysis. The mean ± SD of score differences between pre- and post-intervention was analysed to assess the therapeutic effects of aloe vera. When the data provided in the literature were given as the mean ± SD of outcome variables before and after intervention, or the data were provided as the range of variation, an estimate was made using the method described by Cochrane Handbook 5.0.2. Correlation of 0.5 was used to evaluate SD.

The outcomes of the studies were analysed with the RevMan computer program, version 5.4.1 (Copenhagen: The Nordic Cochrane Centre, The Cochrane Collaboration, 2014). The meta-analysis was conducted by measuring the standardised mean difference (SMD) between the groups, along with 95% confidence intervals (CI); p < 0.05 was considered statistically significant. The significance of any variations in the estimates of the treatment effects from the different trials was assessed by means of Cochran’s test for heterogeneity; the heterogeneity was considered significant if p < 0.1. Heterogeneity between the studies was assessed using the I^2^ statistic.^13,14^

Meta-analysis was undertaken where studies of similar comparisons reported the same outcome measures. Standardised mean difference for pain sensation, treatment effect, size of ulcers and therapeutic duration was calculated and compared between the two studied interventions.

Weighted means across the studies were calculated using a fixed effects model. Where statistically significant heterogeneity was detected (p < 0.1), a random-effects model was used to assess the significance of treatment effects.

## Results

### Literature Search and Study Selection

**Fig 1:**
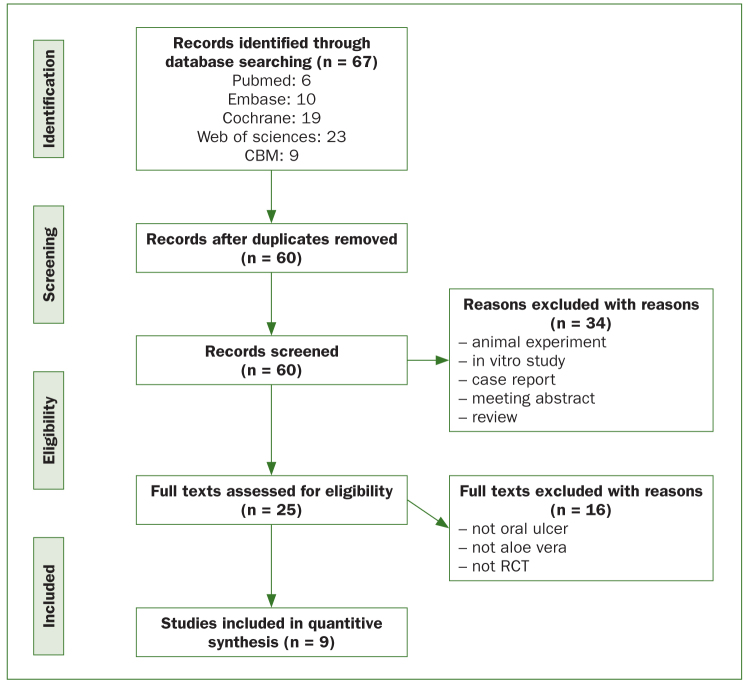
Flow diagram of literature selection process. RCT: randomised controlled trial.

A total of 67 articles were identified through a database search. The articles were compiled using the reference management software NoteExpress; seven duplicate articles were removed. The titles and abstracts of the remaining 60 articles were evaluated; 34 articles were excluded from this systematic review because of nonconformity with the inclusion criteria. When the full texts of the remaining 25 articles were examined, some were again excluded owing to the absence of RCT design, the main disease not being oral ulcers, or the treatment not being aloe vera. Finally, 9 studies were selected for the current meta-analysis (Fig 1).

### General Characteristics of the Included Studies

Characteristics of the included studies are shown in Table 1. Studies were published between 1998 and 2019, comprising 847 subjects. Most of the studies were conducted on adult populations and on both males and females. Two studies were conducted in Iran,^3,20^ two in China,^8,9^ one in India,^11^ one in Saudi Arabia,^18^ one in Japan,^16^ one in Thailand,^4^ and one in the USA.^10^ All of the studies were randomised clinical trials with parallel designs, and all of the studies had at least two groups, in which the effects of aloe vera gel were evaluated in comparison to other medications (n = 8)^4,8-11,16,18,20^ or placebo (n = 1).^3^ The duration of the studies varied from five days to three months.

### Clinical Parameters

The outcome measures reported in the included studies along with the methods of evaluation were highly variable (Table 1). The objective outcomes included size of ulcers, therapeutic duration, skin-patch test and blood test. Size of ulcers was the most frequently measured objective outcome across all studies; five studies assessed objective changes in size of ulcers. Therapy duration was measured in three studies. With respect to the subjective outcomes, mucosal pain was evaluated in seven studies which used the visual analogue scale (VAS), a well-known, validated pain scale.

### Quality of the Included Studies

**Fig 2:**
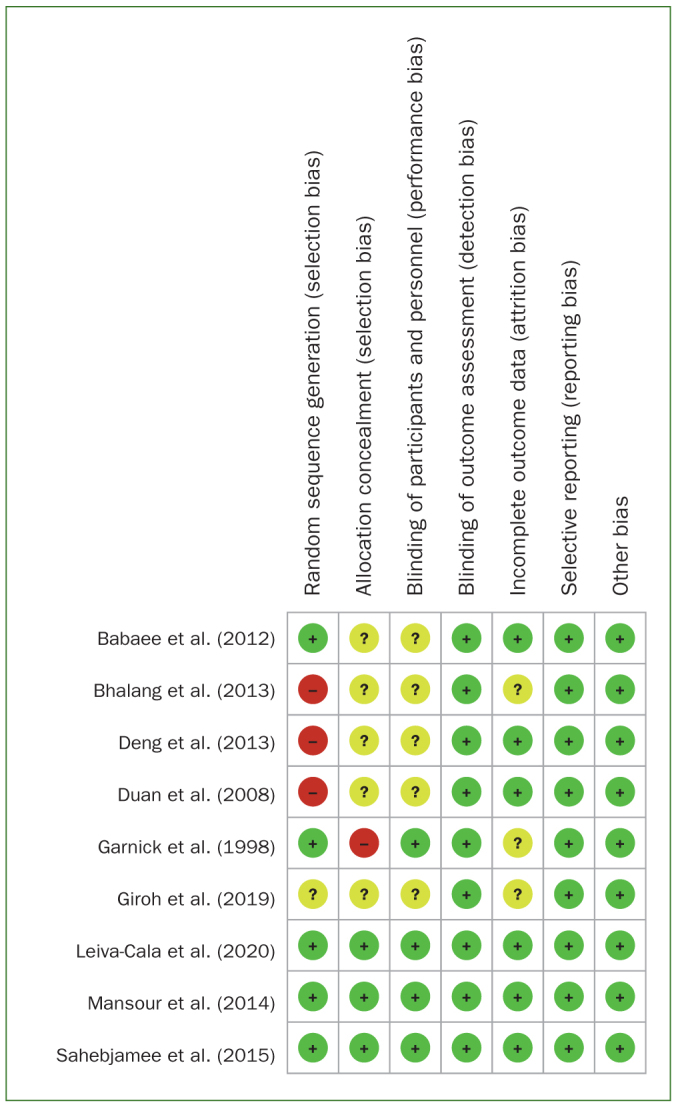
Summary of assessment for risk of bias.

The quality of the included literature was assessed using the Cochrane risk of bias assessment tool. Risk of bias is summarised in Fig 2. Three studies^16,18,20^ had a high risk of bias in random sequence generation, three studies^16,18,20^ had a low risk of bias in allocation concealment, three studies^4,8,9^ did not describe the sequence generation, and one^10^ did not describe allocation concealment, which had a high risk of selection bias. There were 5 studies^3,4,8,9,11^ with unknown risk of implementation bias, and 1 study^10^ that did not have complete outcome data and had a high risk of bias for attrition. The remaining five trials^3,10,16,18,20^ were blinded, four of them^3,10,16,18^ had double-blind study designs, and one of them^20^ had a triple-blind study design; overall, the risk of both reporting and detection bias was low. All studies were feasible protocol studies with consistent primary and secondary outcomes for protocol and study outcomes.

### Study Outcomes

#### Therapeutic effect

Two studies^8,9^ showed a significant improvement in therapeutic effect, which means there was a positive trend in disease treatment in favor of the aloe vera group compared to the control group, promoted quick healing of oral ulcers. One study^16^ suggested that aloe vera gel administration in patients with fixed orthodontic appliances could be important for effective prevention of traumatic oral ulceration.

**Fig 3:**
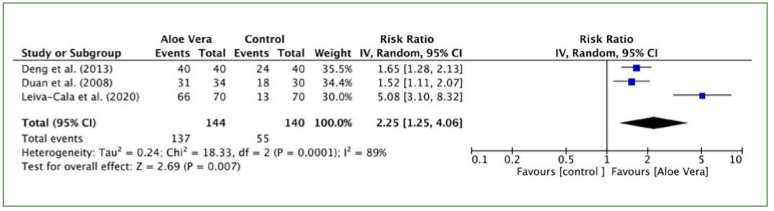
Forest plot for clinical reaction.

The results of the meta-analysis by random effects showed that the efficacy of the aloe vera group was statistically significantly different from that of the control group. The pooled data of three trials^8,9,16^ suggested the clinical efficacy of the aloe vera group was better than that of the control group (I^2^ = 89%, p = 0.007; RR: 2.25, 95% CI: 1.25, 4.06) (Fig 3).

#### Size of ulcers and pain sensation

Five studies reported the size of ulcers and seven studies reported pain sensation. In two studies,^4,11^ topical triamcinolone acetonide 0.1% was found to be more effective than aloe vera in wound healing (as measured by the diameter of ulcer). In contrast, aloe vera gel yielded a better response in terms of pain and burning sensation. One study^3^ showed the duration of complete wound healing, pain score, wound size and inflammation zone diameter in the group treated with aloe vera were statistically significantly lower/smaller than in the control group (p ≤ 0.05) at specific time points after treatment. One study^18^ suggested that aloe vera was superior in decreasing ulcer size, erythema, and exudation. One study^10^ did not demonstrate any consistent effectiveness for the gel containing allantoin, aloe vera, and silicon dioxide on ulcers in the oral cavity, and two studies^8,9^ suggested that aloe vera gel can reduce pain in patients with recurrent oral ulcers.

**Fig 4:**
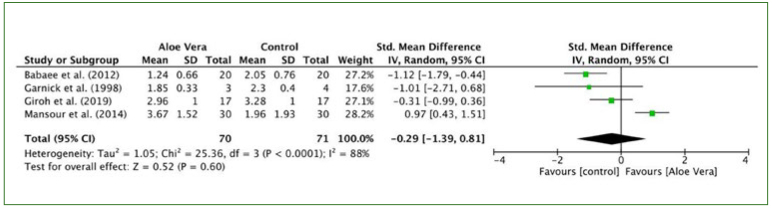
Forest plot for size of ulcers.

The pooled data of four trials^3,10,11,18^ showed no statistically significant differences in the size of ulcers between aloe vera and control groups (I^2^ = 88%, p = 0.60; SMD: -0.29, 95% CI: -1.39, 0.81) (Fig 4).

**Fig 5:**
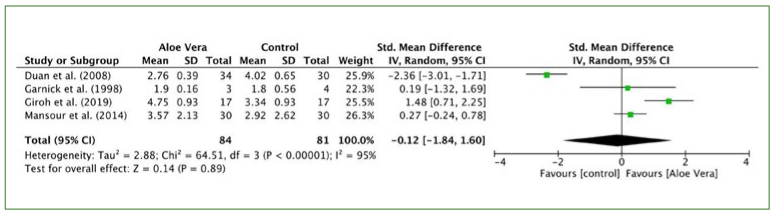
Forest plot for visual analog scale (pain).

The pooled data of four trials^9-11,18^ showed no statistically significant differences in pain sensation between aloe vera and control groups (I^2^ = 95%, p = 0.89; SMD: -0.12, 95% CI: -1.84, 1.60) (Fig 5).

#### Therapy duration

**Fig 6:**
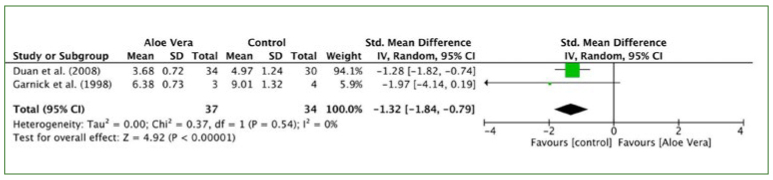
Forest plot for therapeutic duration.

The results of the meta-analysis by fixed effects showed that the efficacy of the aloe vera group was statistically significantly different from that of the control group. The pooled data of two trials^9,10^ suggested the therapy duration of the control group was better compared to the aloe vera group (I^2^ = 0%, p < 0.001; SMD: -1.26, 95% CI: -1.78, -0.74) (Fig 6).

## Discussion

As a general disease whose main symptom is ulcers in mucous membranes, oral ulcers are caused by various factors, including local irritation, systemic diseases, immune factors, etc. The local treatment of oral ulcers incorporates anti-inflammatory, analgesic, and wound-healing medications, as well as systemic administration utilising glucocorticoids and immunologically active medication.^17^ Aloe vera as an herbal medicine is used against many diseases to promote wound healing, and decrease pain and inflammation, also in cases of oral ulcers.^2,16,19^ Based on this rationale, the present study reviewed the previous clinical trials for known clinical therapeutic efficacy of aloe vera on oral ulcers.

The results of the present meta-analysis showed that the treatment effect of aloe vera on patients with oral ulcers was better than that of conventional drugs, which was consistent with results reported by Babaee et al.^3^ The following points may explain this. First, aloe vera promotes epithelial cell migration. It has been found that the topical application of aloe vera increases the synthesis of hyaluronic acid and dermatan sulfate in the granulation tissue of the wound, promoting wound healing. Second, aloe vera increases the wound closure rate and tensile strength of the wound. Aloe vera gel forms a protective coating on the affected areas and helps in healing wounds, accelerates the healing rate, and relieves pain. In addition, aloe vera inhibits the cyclooxygenase pathway and reduces prostaglandin E2 production from arachidonic acid, which contributes to its anti-inflammatory properties.^11^ At the same time, the healing time of the aloe group was shorter than that of the control group; this is also consistent with results from animal studies, which suggested that aloe vera may accelerate the wound healing process.^7^ Nevertheless, the results of the pooled data revealed that aloe vera was as effective as other treatment modalities in clinical relief of pain and the ability to shrink ulcers, with insignificant differences between the groups. Aloe vera, a traditional Chinese medicine, is safer and has few side-effects compared to other interventions. Therefore, due to its analgesic and anti-inflammatory effects, aloe vera is expected to become an alternative medicine for treating oral ulcers, while other conventional therapies have significant side-effects.^1^

Despite precautions, there were still several limitations of our meta-analysis. First of all, it is possible that one article might have been missed as the full text was not obtainable, or because several articles lacked sufficient data, this may have led to bias. Second, due to the high heterogeneity of outcomes in the included studies and insufficient data in some included studies, we could not pool all included publications in our meta-analysis. Moreover, the VAS scores were subjective assessments, which might have influenced the meta-analysis. Finally, we selected the most common oral ulcers, e.g. recurrent aphthous stomatitis, traumatic oral ulceration and radiation mucositis; the included studies included oral ulcers of multiple types, which might have caused heterogeneity in the current results.

Although aloe vera has demonstrated promising therapeutic effects to promote healing and reduce the pain of oral ulcers, there are still many problems to be solved, e.g. the optimal application of aloe vera, unclear treatment mechanisms, the possible side-effects of aloe vera, etc. This meta-analysis may provide orientation for further exploration of clinical applications of aloe vera in the treatment of oral ulcers.

## Conclusion

As a promising medicine to promote the healing of oral ulcers, aloe vera has demonstrated better therapeutic effects and shorter healing times than other medications or placebo. Furthermore, it possessed the same effects in relieving pain and reducing ulcer size as other interventions, but it has higher safety and almost no side-effects. Therefore, the results of this meta-analysis should be further confirmed by higher quality, multicenter clinical trials with larger sample sizes.
